# The real face of LiF

**DOI:** 10.1093/nsr/nwaf448

**Published:** 2025-10-20

**Authors:** Hongtao Qu, Xikun Zhang, Bao-Lian Su

**Affiliations:** Laboratory of Inorganic Materials Chemistry (CMI), University of Namur, Belgium; Laboratory of Inorganic Materials Chemistry (CMI), University of Namur, Belgium; Laboratory of Inorganic Materials Chemistry (CMI), University of Namur, Belgium; State Key Laboratory of Advanced technology for Materials Synthesis and Processing, Wuhan University of Technology, China

The solid–electrolyte interphase (SEI) is an important component in lithium-ion batteries (LIBs), formed *in situ* by electrolyte reduction on the anode during the initial cycles. It dictates long-term battery stability by blocking electrons to prevent continuous electrolyte decomposition, while allowing lithium ions to migrate freely. Structurally, the SEI is a complex, disordered, nanoscale passivation layer, posing major challenges for characterization. Given its critical role, extensive efforts have been devoted to identifying its key components and correlating them with battery performance and failure mechanisms [1].

The widely accepted SEI composition includes LiF, Li_2_O, and Li_2_CO_3_ as principal inorganic constituents, together with lithium alkyl carbonates in carbonate-based electrolytes and lithium alkoxides in ether-based systems as the dominant organic species. Among them, LiF is often regarded as indispensable, regulating ion transport and suppressing dendrite growth due to its wide electrochemical stability window and high mechanical strength [2]. Previous studies have shown that LiF additives in electrolytes promote uniform lithium deposition/dissolution and extend the lifetime of lithium-metal batteries (LMBs) [3]. Numerous reports highlight the advantages of LiF-rich SEIs in facilitating lithium-ion transport [4]. Yet, this observation is seemingly at odds with the intrinsic electronic and ionic insulating nature of bulk LiF. Recent work demonstrates that nanometric LiF exhibits unexpectedly high ionic conductivity with a reduced diffusion barrier, underscoring that the role of LiF within the SEI (LiF_SEI_) remains incompletely understood [5].

**Figure 1.**
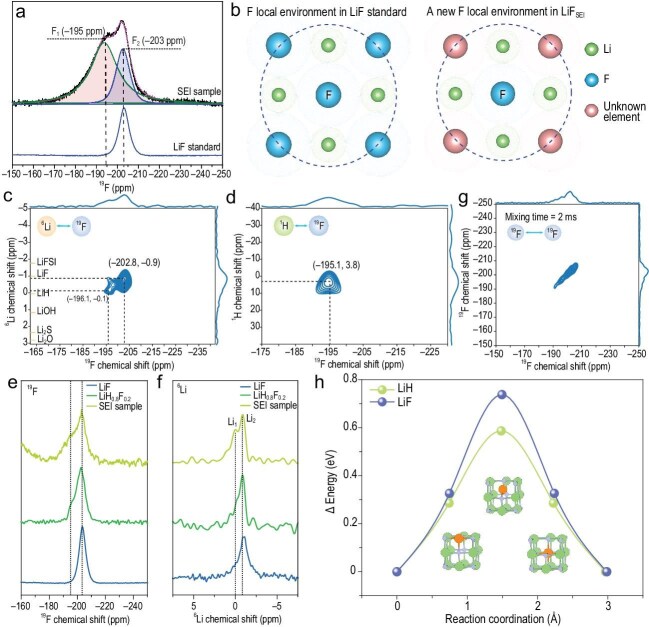
(a) ^19^F MAS NMR spectrum of SEIs. (b) F local environment in LiF and a new F-containing species. (c) ^6^Li–^19^F HETCOR spectrum of SEI sample. (d) ^1^H–^19^F HETCOR spectrum of SEI sample. (e, f) Comparison of 1D ^19^F NMR spectra and ^19^F → ^6^Li CP spectra of LiF standard, LiH_0.8_F_0.2_ standard, and LiF_SEI_. (g) 2D ^19^F–^19^F radio-frequency-driven recoupling (RFDR) spectrum of SEI sample. (h) Theoretical calculation of Li ion diffusion barrier in LiF and LiH. Adapted with permission from ref. [6].

Writing in *Nature*, Yuxuan Xiang, Yun Song, Yizhou Zhu and colleagues provide new insight into the fine structure of LiF in SEIs using non-destructive, element-specific, and local-sensitive solid-state NMR spectroscopy [6]. They reveal that LiF_SEI_ is heterogeneous, comprising limited LiF–LiH solid solutions: a hydrogen-rich phase (LiH_1−y_F_y_) and a fluorine-rich phase (LiF_1−x_H_x_). The team’s analysis began with an asymmetrical ^19^F NMR signal from LiF_SEI_, in contrast to the symmetrical resonance of standard LiF (Fig. 1a, b). A second fluorine-containing species (F1, −195 ppm) was identified alongside LiF (F2, −203 ppm). Two-dimensional ^6^Li–^19^F (Fig. 1c) and ^1^H–^19^F (Fig. 1d) heteronuclear correlation (HETCOR) NMR confirmed spatial proximity between Li–F and H–F units, indicating the presence of Li–F–H species. Comparison of ^19^F MAS NMR spectra of LiF_SEI_ with synthesized LiF_1−x_H_x_ standards (x = 0.4, 0.8, 0.9, 0.95) showed the closest agreement with LiH_0.8_F_0.2_ (Fig. 1e), which is also corroborated by ^6^Li MAS NMR (Fig. 1f).

To further explore the role of LiH–LiF solid solutions in battery chemistry, Xiang and co-workers performed 2D ^19^F–^19^F radio-frequency-driven recoupling (RFDR) experiments on LiF_SEI_ (Fig. 1g). No cross-peaks between F_1_ and F_2_ were observed, indicating the LiF_1−x_H_x_ and LiH_1−y_F_y_ phases co-exist in the SEI, which is also confirmed by Synchrotron XRD. Theoretical calculations show that the Li ion diffusion barrier in LiH is lower than in LiF, and increasing hydrogen content in LiF_1−x_H_x_ enhances ionic conductivity (Fig. 1h).

Xiang and co-workers then applied these findings to SEI design. Li-metal electrodes coated with pure LiF or H-rich LiH_1−y_F_y_ were tested in symmetric Li/Li cells at 10 mA cm⁻². Electrodes with LiH_1−y_F_y_ coatings exhibited markedly lower voltage hysteresis and reduced interfacial impedance, demonstrating improved ion transport across the interface.

This work uncovers the heterogeneous nature of LiF in SEIs and identifies, for the first time, coexisting H-rich and F-rich Li–H–F solid solutions, whose presence enhances cycling stability. These findings reshape our understanding of SEI functionality. While SEIs generally contain both inorganic and organic species, most design strategies focus on inorganic-rich compositions for their mechanical robustness, stability, and uniform ion transport. However, recent evidence suggests that inorganic-rich SEIs may hinder low-temperature performance due to high-impedance Li-bearing inorganics. A more nuanced understanding of the roles and synergies among different SEI species is urgently needed.

SEI characterization remains intrinsically challenging, as the layer is nanometers thin, scarce in quantity, and highly sensitive to air. X-ray-based techniques risk altering or destroying native SEI species, while *ex-situ* handling often introduces contamination or loss of key components. Advanced *in situ* and *operando* methods are therefore essential to reveal the true nature of SEIs within functioning batteries. This work also highlights the unique value of solid-state NMR as a non-destructive, element-specific characterization tool [7]. NMR spectroscopy is already widely used to investigate battery systems, from ion dynamics and local structure to interfacial chemistry. Many SEI components contain NMR-active nuclei (^1^H, ^13^C, ^6/7^Li, ^17^O, ^14/15^ N, ^19^F), among which ^1^H, ^7^Li, and ^19^F are especially accessible due to high natural abundance and sensitivity. Probing less sensitive nuclei such as ^13^C, ^17^O, ^14/15^ N, remains challenging, though isotope enrichment and advanced approaches such as dynamic nuclear polarization (DNP) offer promising routes, provided specialized instrumentation is available.

Together, these insights underscore that the SEI remains the most important yet least understood component of rechargeable batteries. Its chemistry is not universal but highly dependent on electrolyte formulation and solvation structure [8]. Future research should focus on exploring the interrelationships among the various components of the SEI, rather than examining individual constituents in isolation. Developing a comprehensive understanding of SEIs across diverse systems will be crucial for the rational design of next-generation, high-energy-density electrochemical storage technologies.
